# Broad-spectrum receptor tyrosine kinase inhibitors overcome *de novo* and acquired modes of resistance to EGFR-targeted therapies in colorectal cancer

**DOI:** 10.18632/oncotarget.26663

**Published:** 2019-02-12

**Authors:** Ramona Graves-Deal, Galina Bogatcheva, Saba Rehman, Yuanyuan Lu, James N. Higginbotham, Bhuminder Singh

**Affiliations:** ^1^ Department of Medicine, Vanderbilt University Medical Center, Nashville, TN, USA; ^2^ Epithelial Biology Center, Vanderbilt University Medical Center, Nashville, TN, USA

**Keywords:** cetuximab resistance, colorectal cancer, EGFR, RTK inhibition, MET

## Abstract

It is increasingly appreciated that 3D cultures are more predictive of *in vivo* therapeutic efficacy than 2D cultures. Using *in vitro* 3D type I collagen cultures of human colorectal cancer (CRC) cell line HCA-7 derivatives CC, SC, and CC-CR, we previously identified that activation of receptor tyrosine kinases (RTKs) MET and RON contributed to resistance to the EGF receptor (EGFR)-directed therapeutic antibody cetuximab. The *de novo* mode of cetuximab resistance in SC cells could be overcome by crizotinib, a multi-RTK inhibitor that also targets MET and RON. We now show that crizotinib also overcomes acquired cetuximab resistance in CC-CR cells. Phospho-RTK array analysis showed increased phosphorylation of several RTKs, including MET and RON, in SC and CC-CR cells compared to cetuximab-sensitive CC counterparts. Furthermore, other multi-RTK inhibitors cabozantinib and BMS-777607 helped overcome cetuximab resistance, as measured by 3D colony growth and activation state of key signaling molecules. Conversely, addition of RTK ligands HGF and NRG1 induced cetuximab resistance in CC cells, which could be blocked by addition of crizotinib. We further determined the mechanism of the cooperative effect of cetuximab and crizotinib by FACS analysis and observed increased cell cycle arrest in G1 phase in cetuximab-resistant CRC 3D cultures. Finally, we show that crizotinib overcomes cetuximab resistance *in vivo* in SC nude mice xenografts. Thus, our work shows that multi-RTK inhibition strategy is a potent, broadly applicable strategy to overcome resistance to EGFR-targeted therapeutics in CRC and highlights the relevance of 3D cultures in these studies.

Statement of implication: Using *in vitro* 3D CRC cultures and *in vivo* CRC xenografts, we show that parallel inhibition of multiple RTKs with small molecule inhibitors overcomes *de novo* and acquired resistance to EGFR-directed therapies in CRC.

## INTRODUCTION

Receptor tyrosine kinase (RTK) signaling is one of the major dysregulated pathways in cancer that contributes to transformation and is a major therapeutic target [1–3]. In colorectal cancer (CRC), the RTK EGFR is overexpressed in more than 50% of cases and is linked to poor prognosis and metastasis [4]. EGFR-targeting monoclonal antibodies, cetuximab and panitumumab, are approved by the U.S. FDA for the treatment of individuals with advanced wild-type *KRAS* CRC [5–8]. Cetuximab use is contraindicated with *KRAS* mutations, which lead to constitutive activation of downstream signaling, rendering EGFR-directed therapies ineffective [8, 9]. KRAS mutations are the most common form of cetuximab resistance and occur in more than 40% of both *de novo* and acquired cases of cetuximab resistance [10, 11]. Other frequent genetic and non-genetic mechanisms of resistance are mutations (*NRAS*, *BRAF*, *EGFR*, *PIK3CA*, and *PTEN*), amplifications (*EGFR*, *ERBB2*, and *MET*), and overexpression (TGFA, AREG, VEGF, HGF, MIR100HG) or MET/RON activation [12–21]. Thus, in advanced CRC in particular, there is a need to enhance the effectiveness of clinically approved targeted therapies (cetuximab, panitumumab, bevacizumab, and ramucirumab; Supplementary Table 1) and prevent or overcome emergence of resistance to these therapies.

During our research, we have shown that normal EGFR signaling, regulated by the targeted availability of its ligands, is essential for normal epithelial function, and its dysregulation may lead to transformation [22–24]. Our work has benefited from our observations that *in vitro* 3D cultures better recapitulate *in vivo* conditions than the prevalent, 2D plastic cultures. We established a novel 3D culture system that identified key disease-relevant genes in CRC [21]. By culturing a CRC cell line, HCA-7, in 3D type I collagen, we have generated two cell lines (CC and SC) with distinct morphological, genetic, biochemical, and functional properties. CC form polarized cystic colonies in 3D, while SC form spiky colonies. CC are cetuximab sensitive, while SC are cetuximab resistant in 3D. On plastic, both lines are morphologically indistinguishable, and both are resistant to cetuximab [21]. We also observed increased tyrosine phosphorylation of MET and RON in SC cells. Moreover, we show that SC cetuximab resistance can be overcome by addition of the dual MET/RON tyrosine kinase inhibitor crizotinib. We also generated cetuximab-resistant CC derivatives and termed them CC-CR [20].

In this report, we show that the multi-RTK inhibition strategy overcomes both *de novo* and acquired modes of resistance to EGFR-directed therapies. Using SC and CC-CR cells, we show that the efficacy of multiple EGFR-directed therapeutic antibodies (cetuximab, panitumumab, and MM-151) can be enhanced by addition of small molecule RTK inhibitors (crizotinib, cabozantinib, and BMS-777607). Moreover, we also identified that activation of the RTKs by addition of their cognate ligands induces cetuximab resistance in the sensitive CC line. We further tested the cetuximab/crizotinib combination *in vivo* and showed that crizotinib addition overcomes cetuximab resistance in SC nude mice xenografts. Thus, RTK inhibition acts cooperatively to enhance effectiveness of EGFR-targeted therapies in CRC.

## RESULTS

### Overcoming *de novo* and acquired modes of cetuximab resistance by RTK inhibition with crizotinib

**Figure 1:**
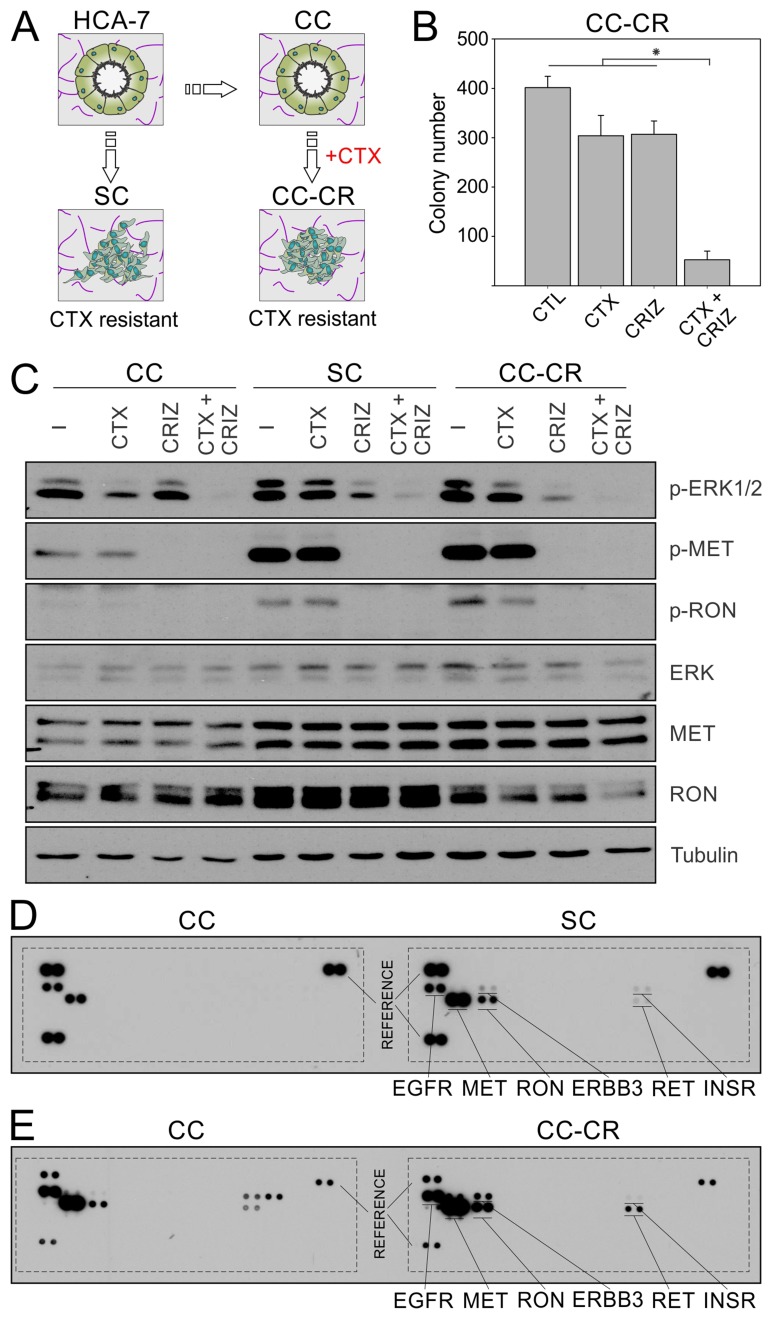
Overcoming *de novo* and acquired mode of cetuximab resistance in CRC by crizotinib (**A**) Parental HCA-7 cells and its subclone, CC, predominately form uniform cysts in 3D collagen cultures, while SC derivatives form disorganized colonies. CC-CR, derived from CC cultured in 3D in the presence of cetuximab, also form disorganized colonies with incompletely cleared lumens. Both SC and CC-CR are cetuximab-resistant and exhibit high levels of MET and RON phosphorylation. (**B**) Two thousand CC-CR cells were cultured in type I collagen for two weeks in the presence of cetuximab (CTX, 3 μg/ml) and/or crizotinib (CRIZ, 0.05 µM). Colony counts are plotted as mean ± SEM. ^*^indicate statistically significant differences (*p* < 0.05). (**C**) One hundred thousand CC, SC, and CC-CR cells were cultured in type I collagen for seven days and incubated with cetuximab (CTX, 3 μg/ml) and/or crizotinib (CRIZ, 0.05 µM) for 6 h. Middle collagen layers containing cells were lysed, resolved on SDS-PAGE, and immunoblotted for the indicated proteins. Please note that reduction in ERK1/2 phosphorylation by cetuximab is only achieved in CC cells (compare lanes 2, 6, and 10 with lanes 1, 5, and 9, respectively). (**D**) CC and SC 3D cultures were lysed, and equal amounts of cell lysates were analyzed using the human phospho-RTK array kit; CC RTK phosphorylation status is on the left, while SC RTK phosphorylation status is on the right, with key RTKs highlighted. Three reference pairs are on each phospho-RTK blot of CC and SC. (**E**) Comparison of CC (left) and CC-CR (right) tyrosine phosphorylation status of individual RTKs; samples were processed as in D.

Previously, we established three lines from the CRC line HCA-7 by seeding the cells in 3D in type I collagen as single cell suspension. These three lines are 1) CC, which are sensitive to cetuximab, 2) SC, which are spontaneously resistant to cetuximab, and 3) CC-CR, which were derived by culturing CC cells in the presence of cetuximab (Figure 1A). Collectively, SC and CC-CR represent *de novo* and acquired modes of cetuximab resistance, respectively [20, 21]. We previously showed that *de novo* mode of cetuximab resistance in SC cells could be overcome by addition of the multi-RTK inhibitor crizotinib [21]. We also showed upregulation MET and RON phosphorylation in SC cells compared to CC, which could be inhibited by addition of crizotinib. In this report, we tested if acquired mode of cetuximab resistance in CC-CR cells could be overcome by addition of crizotinib. Cetuximab or crizotinib alone were unable to significantly reduce colony number in CC-CR 3D cultures; the combination, however, markedly inhibited CC-CR colony growth (Figure 1B). Thus, crizotinib is able to overcome both *de novo* and acquired modes of cetuximab resistance in the 3D CRC culture system.

Next, we compared CC, SC, and CC-CR at the signaling level. In addition to increased MET and RON phosphorylation in SC cells shown previously, we observed similar upregulation of MET and RON phosphorylation in CC-CR cells compared to their cetuximab sensitive counterpart, CC cells (Figure 1C). Crizotinib addition decreased MET and RON tyrosine phosphorylation in all three lines. However, at the downstream signaling level, reduction in pERK1/2 levels was more pronounced in cetuximab-resistant SC and CC-CR cells only; pERK1/2 levels in CC cells remained largely unperturbed after crizotinib addition. Conversely, in response to cetuximab addition, the reduction in pERK1/2 levels was most pronounced in CC cells; SC and CC-CR were largely unaffected at pERK1/2 levels. These results indicate that ERK1/2 activity is largely dependent on upstream EGFR activity in CC cells, whereas it is dependent on other RTKs in cetuximab-resistant SC and CC-CR cells. The combination treatment, however, was most effective in all three cell lines in reducing ERK1/2 phosphorylation levels.

We next tested the global RTK phosphorylation levels of SC and CC-CR and compared with the cetuximab-sensitive CC counterparts. The major RTKs with elevated tyrosine phosphorylation in SC and CC-CR compared to CC were MET, RON, ERBB3, RET, and INSR (Figure 1D, 1E) (array spotting schematic in Supplementary Figure 1).

### Overcoming panitumumab resistance in SC and CC-CR cells by crizotinib

**Figure 2:**
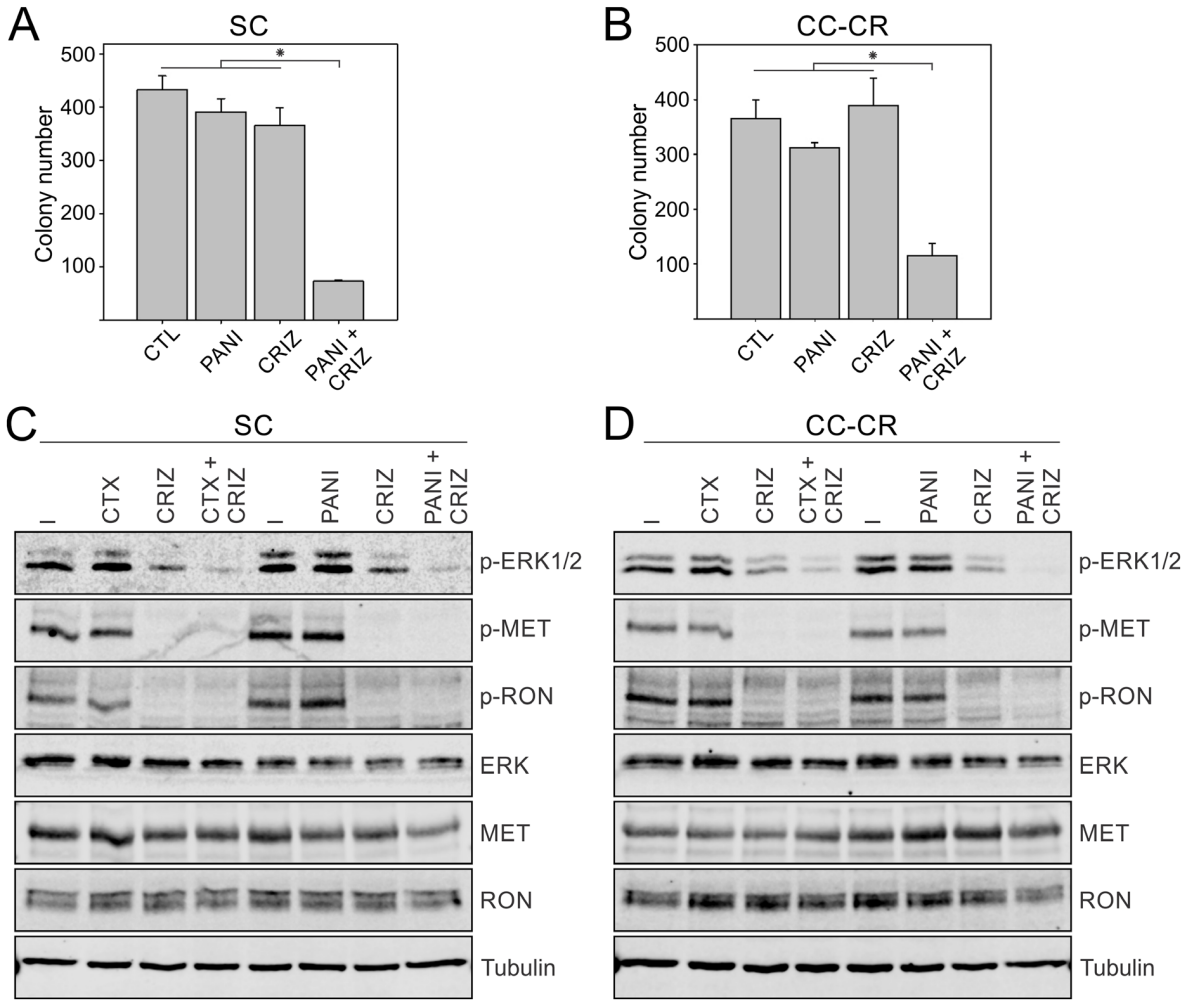
Overcoming panitumumab resistance in SC and CC-CR cells by crizotinib Two thousand SC (**A**) and CC-CR (**B**) cells were cultured in type I collagen for two weeks. Fresh medium was added with panitumumab (PANI, 3 μg/ml) and crizotinib (CRIZ, 0.05 µM) every two to three days as indicated. Colony counts are plotted as mean ± SEM. ^*^indicate statistically significant differences (*p* < 0.05). (**C, D**) One hundred thousand SC (C) and CC-CR (D) cells were cultured in type I collagen for seven days and incubated with cetuximab (CTX, 3 μg/ml), panitumumab (PAN, 3 μg/ml), and/or crizotinib (CRIZ, 0.05 µM) for 6 h. Middle collagen layers containing cells were lysed, resolved on SDS-PAGE, and immunoblotted for the indicated proteins.

In contrast to cetuximab, panitumumab is a fully humanized monoclonal antibody that also targets EGFR and is approved by U.S. FDA for treating KRAS wild-type advanced CRC. Both SC and CC-CR were resistant to panitumumab in 3D. However, in both SC and CC-CR cells, the panitumumab/crizotinib combination treatment led to significant reduction in colony number compared to either treatment alone (Figure 2A, 2B). At the signaling level, both SC and CC-CR responded to the drug combination, as ERK1/2 phosphorylation was markedly reduced in the panitumumab/crizotinib combination treatment group in both SC and CC-CR cells as compared to either inhibitor alone (Figure 2C, 2D). Near complete inhibition of MET and RON phosphorylation in SC and CC-CR cultures indicates drug efficacy (Figure 2C, 2D). Combined, these results show that lines resistant to cetuximab are also resistant to panitumumab, and that panitumumab resistance may be overcome by crizotinib treatment.

### Overcoming MM-151 resistance in SC and CC-CR cells by crizotinib

**Figure 3:**
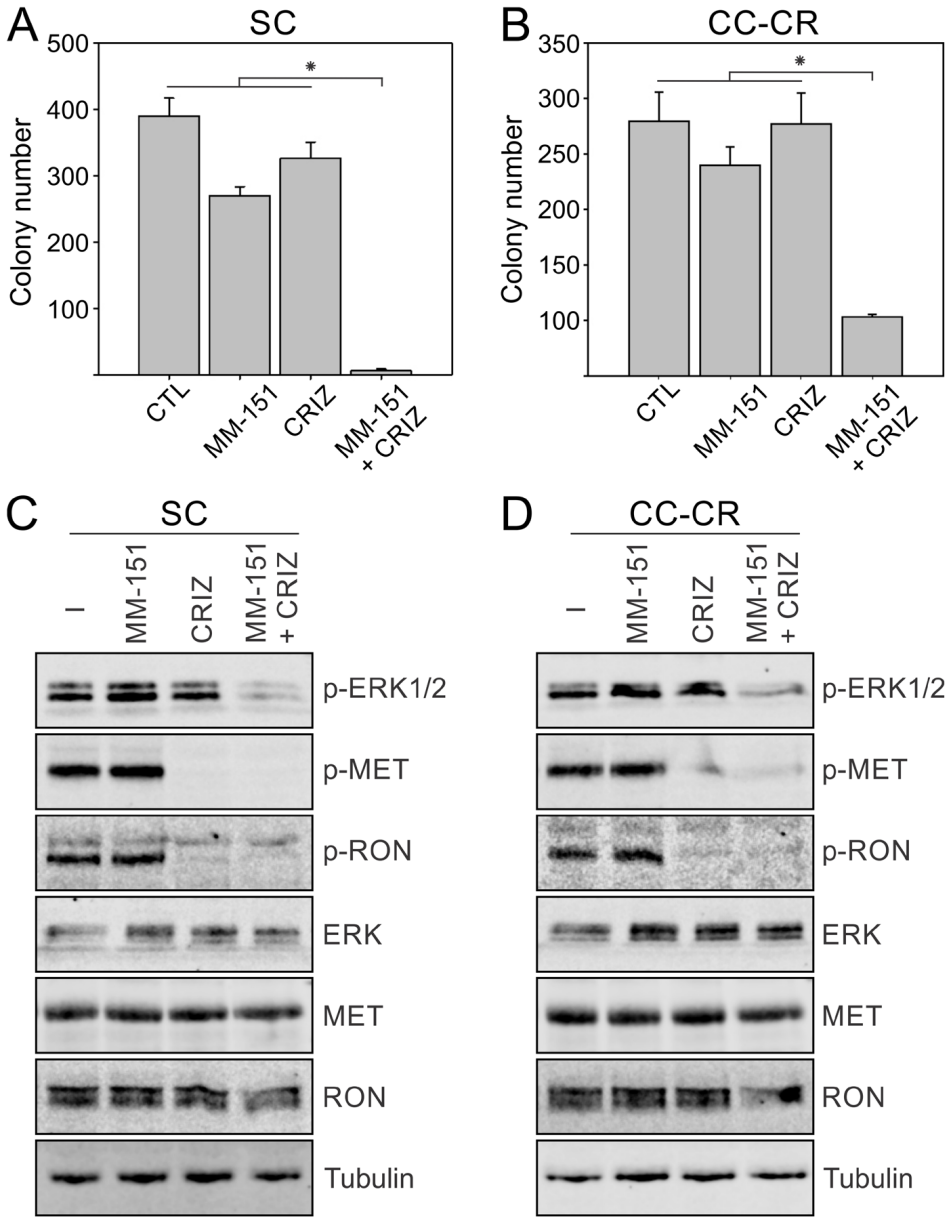
Overcoming MM-151 resistance in SC and CC-CR cells by crizotinib Two thousand SC (**A**) and CC-CR (**B**) cells were cultured in type I collagen for two weeks. Fresh medium was added with MM-151 (3 μg/ml) and crizotinib (CRIZ, 0.05 µM) every two to three days as indicated. Colony counts are plotted as mean ± SEM. ^*^indicate statistically significant differences (*p* < 0.05). (**C, D**) One hundred thousand SC (C) and CC-CR (D) cells were cultured in type I collagen for seven days and incubated with cetuximab (CTX, 3 μg/ml), MM-151 (3 μg/ml), and/or crizotinib (CRIZ, 0.05 µM) for 6 h. Middle collagen layers containing cells were lysed, resolved on SDS-PAGE, and immunoblotted for the indicated proteins.

In contrast to cetuximab and panitumumab, which bind to a single epitope in EGFR extracellular domain, MM-151 is a mixture of three fully human monoclonal antibodies that bind distinct regions in the EGFR extracellular domain [25]. MM-151 has been shown to overcome acquired resistance to cetuximab and panitumumab in CRC harboring EGFR extracellular domain mutations [26]. In our previous reports, we have shown that EGFR is wild-type in both SC and CC-CR cells [20, 21]. Both SC and CC-CR were resistant to MM-151 in 3D (Figure 3A, 3B). However, addition of crizotinib again cooperated with MM-151 in inhibiting SC and CC-CR colony growth (Figure 3A, 3B). At the signaling level, both SC and CC-CR cultures exhibited cooperative reduction of ERK1/2 phosphorylation by the MM-151/crizotinib combination (Figure 3C, 3D). Combined, these results show that, like cetuximab and panitumumab, MM-151 is unable to inhibit SC and CC-CR colony growth, and that MM-151 resistance can be overcome by addition of crizotinib.

### Overcoming resistance to cetuximab by multi-RTK inhibitors cabozantinib, BMS-777607, and bemcentinib (BGB324)

**Figure 4:**
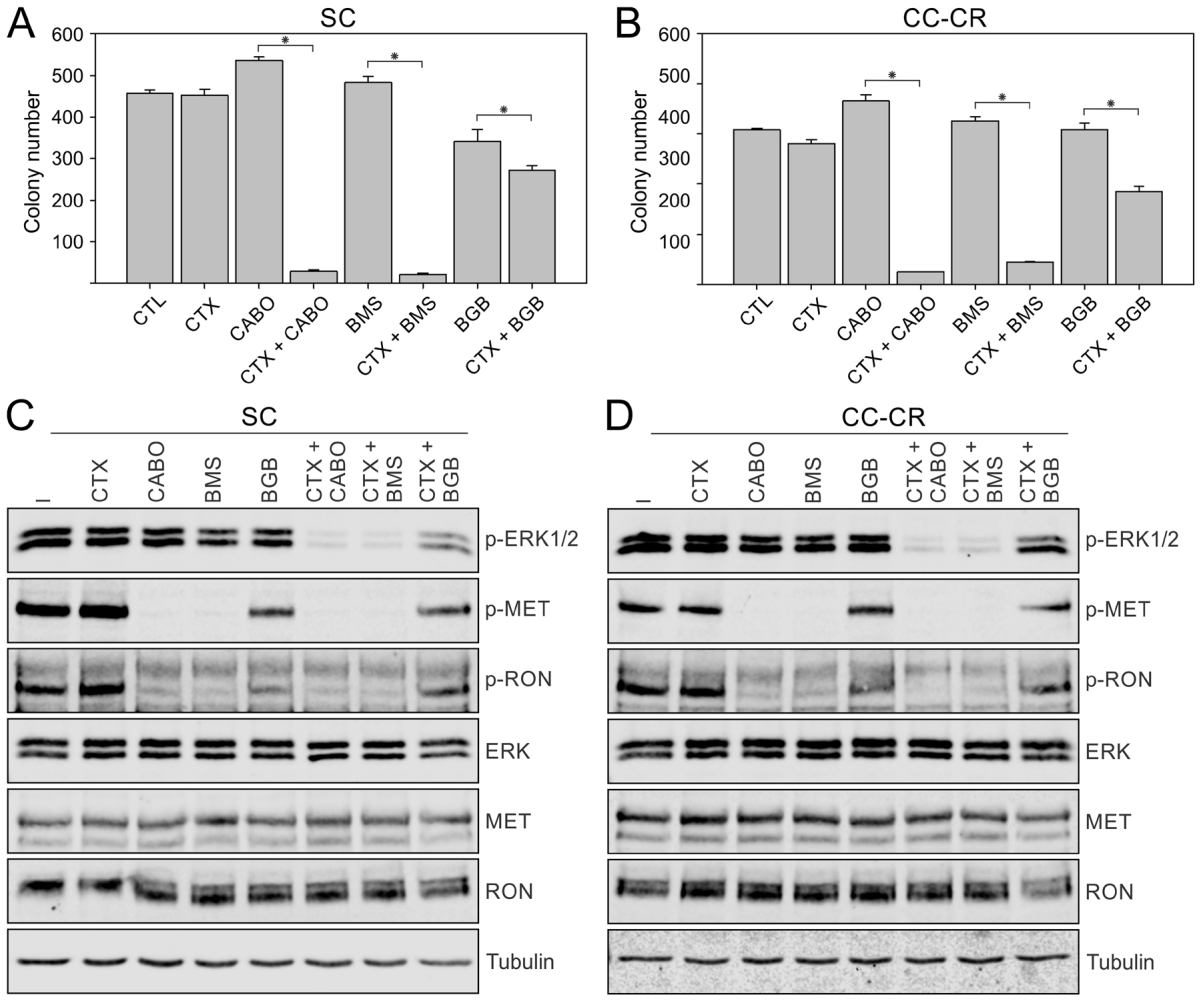
Overcoming cetuximab resistance in SC and CC-CR cells by multi-RTK inhibitors Two thousand SC (**A**) and CC-CR (**B**) cells were cultured in type I collagen for two weeks. Fresh medium was added with cetuximab (CTX, 3 μg/ml), cabozantinib (CABO, 1.5 μM), BMS-777607 (BMS, 1.5 μM), and/or bemcentinib/BGB324 (BGB, 1.8 µM) every two to three days as indicated. Colony counts are plotted as mean ± SEM. ^*^indicate statistically significant differences (*p* < 0.05). (**C, D**) One hundred thousand SC (C) and CC-CR (D) cells were cultured in type I collagen for seven days and incubated with cetuximab (CTX, 3 μg/ml), cabozantinib (CABO, 1.5 μM), BMS-777607 (BMS, 1.5 μM), and/or bemcentinib/BGB324 (BGB, 1.8 µM) for 6 h. Middle collagen layers containing cells were lysed, resolved on SDS-PAGE, and immunoblotted for the indicated proteins.

We next tested if other RTK inhibitors can effectively replace crizotinib in the cetuximab/crizotinib combination treatment. For this, we used three other RTK inhibitors with overlapping targeting capacities. Cabozantinib and BMS-777607 target the RTKs MET and RON with high affinity, whereas the preferred target for bemcentinib is AXL [27]. In both SC and CC-CR cells, cabozantinib and BMS-777607 addition alone did not induce significant cytotoxicity; colony numbers were comparable to untreated controls (Figure 4A, 4B). However, with bemcentinib alone significantly less colonies were observed in SC cultures (Figure 4A). Up to 80–95% less colonies were observed in cetuximab/cabozantinib or cetuximab/BMS-777607 combinations compared to single agents or untreated controls in SC and CC-CR cultures (Figure 4A, 4B). At the signaling level, both cabozantinib and BMS-777607 were potent inhibitors of MET and RON phosphorylation in both SC and CC-CR cells, whereas bemcentinib was unable to induce appreciable reduction in MET/RON phosphorylation at a similar concentration (Figure 4C, 4D). In the combination treatments, cetuximab/cabozantinib and cetuximab/BMS-777607 effectively reduced ERK1/2 phosphorylation levels, while cetuximab/bemcentinib combination led to modest reduction in ERK1/2 phosphorylation. Combined together, these results indicate that cabozantinib and BMS-777607 can overcome *de novo* and acquired resistance to cetuximab in CRC lines.

### Inducing cetuximab resistance in CC cells by addition of RTK ligands, HGF and NRG1

**Figure 5:**
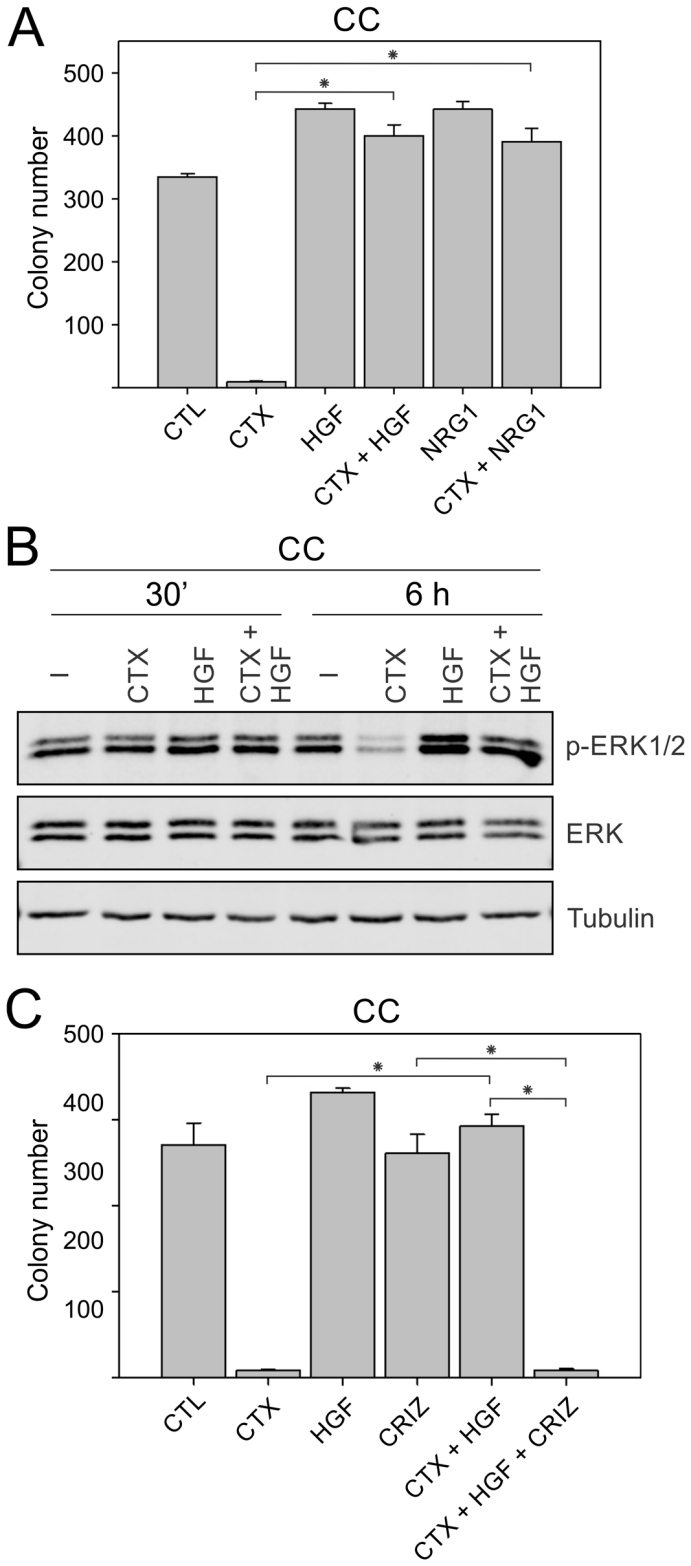
Inducing cetuximab resistance in CC cells by addition of RTK ligands, HGF and NRG1 (**A**) Two thousand CC cells were cultured in type I collagen for two weeks. Fresh medium was added with cetuximab (CTX, 3 μg/ml), HGF (50 ng/ml), or NRG1 (50 ng/ml) every two to three days as indicated. Colony counts are plotted as mean ± SEM. ^*^indicate statistically significant differences (*p* < 0.05). (**B**) One hundred thousand CC cells were cultured in type I collagen for seven days and incubated with cetuximab (CTX, 3 μg/ml), HGF (50 ng/ml), or NRG1 (50 ng/ml) for 6 h. Middle collagen layers containing cells were lysed, resolved on SDS-PAGE, and immunoblotted for the indicated proteins. (**C**) Two thousand CC cells were cultured in type I collagen for two weeks. Fresh medium was added with cetuximab (CTX, 3 μg/ml), HGF (50 ng/ml), and/or crizotinib (CRIZ, 0.05 μM) every two to three days as indicated. Colony counts are plotted as mean ± SEM. ^*^indicate statistically significant differences (*p* < 0.05).

Using multiple anti-EGFR antibodies and multi-RTK inhibitors above, we showed that cetuximab resistance in SC and CC-CR cells may be overcome by RTK inhibition. Now, using cetuximab-sensitive CC counterparts, we next tested if RTK activation may impart cetuximab resistance. Since we found upregulation of MET, RON, ERBB3, INSR, and RET tyrosine phosphorylation in cetuximab-resistant SC and CC-CR cells compared to CC, we elected to activate two of these RTKs, MET and ERBB3, by addition of their cognate ligands, HGF and NRG1, respectively. Addition of cetuximab to CC cultures led to more than 95% reduction in colony counts (Figure 5A). In line with their growth factor properties, HGF or NRG1 addition led to slight increase in colony number (Figure 5A). However, when added along with cetuximab, both HGF and NRG1 were able to block cetuximab-mediated growth inhibition of CC colonies (Figure 5A). Among NRG family members, this effect seems to be specific to NRG1, as NRG2 tested at the same concentration failed to provide protection against cetuximab (Supplementary Figure 2). NRG3, which binds to HER4 instead of ERBB3, is similarly ineffective in providing protection against cetuximab (Supplementary Figure 2). We next tested the effect of HGF addition at the signaling level and found that cetuximab addition alone was able to inhibit ERK1/2 phosphorylation in CC 3D cultures within 6 hours (Figure 5B). HGF addition increased ERK1/2 levels, which could not be inhibited by cetuximab at the 6 h time point (Figure 5B). We next tested if the cetuximab-protective effect of HGF could be overcome by inactivation of its cognate receptor MET by crizotinib. In the combined cetuximab/HGF/crizotinib treatment, CC colony growth was largely inhibited, similar to cetuximab treatment alone (Figure 5C). Combined together, these results indicate that cetuximab-sensitive CC cells may become cetuximab resistant by activation of RTKs, MET or ERBB3, by addition of their cognate ligands HGF or NRG1.

### Mechanism of clearance of SC and CC-CR cells with multi-RTK inhibition strategy

**Figure 6:**
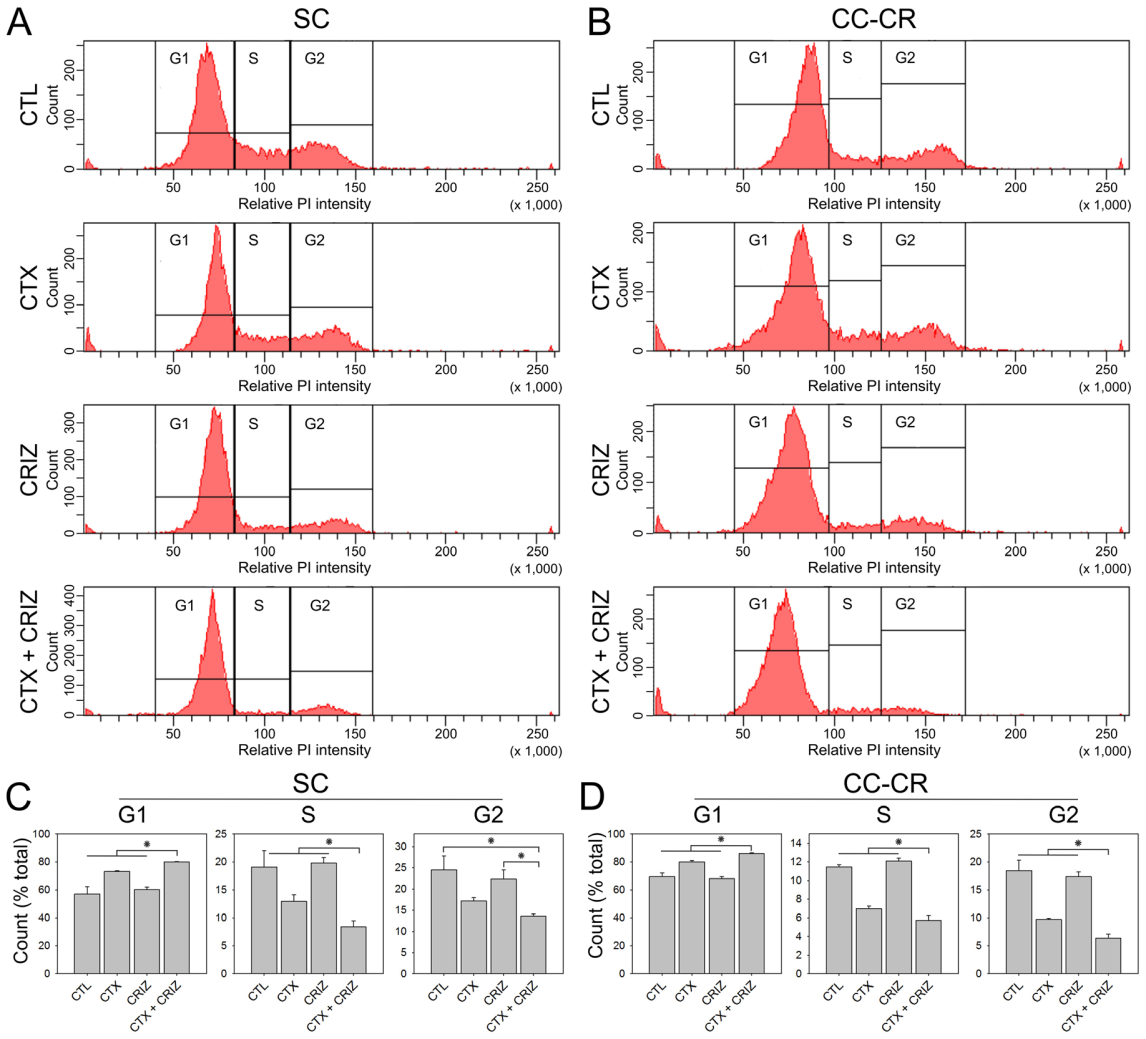
Induction of cell cycle arrest by multi-RTK inhibition in cetuximab-resistant CRC lines (**A, B**) One hundred thousand SC (A) and CC-CR (B) cells were cultured in type I collagen for seven days and incubated with cetuximab (CTX, 3 μg/ml) and/or crizotinib (CRIZ, 0.05 μM) for 20 h, as indicated. Middle collagen layers containing cells were processed and analyzed for nuclear DNA content (propidium iodide, PI) by flow cytometry. The histograms generated show relative PI intensity on the x-axis with the particle count on the y-axis (linear scales). (**C, D**) Graphical representation of SC (C) and CC-CR (D) cells distributed among the G1, S, and G2 phases of cell cycle in left, middle, and right histograms under different treatment conditions. Values are plotted as fractions of total cells counted (mean ± SEM). ^*^indicates statistically significant differences (*p* < 0.05).

To identify the mechanism of SC and CC-CR colony growth inhibition due to multi-RTK inhibition, we elected to perform cell cycle analysis of SC and CC-CR cultures under different treatments. We treated one-week old SC and CC-CR cultures with drug combinations for ∼20 hours and then subjected them to cell cycle analysis by flow cytometry. For both SC and CC-CR cultures, we identified that cetuximab alone induces a small but significant G0/G1 cell cycle block (Figure 6A, 6B). Crizotinib alone was unable to induce G0/G1 cell cycle block. In the cetuximab/crizotinib combination, however, more cells accumulated in the G0/G1 phase of cell cycle, compared to control cells or individual treatments, along with a concomitant reduction in S and G2/M population (Figure 6A–6D).

### Overcoming cetuximab resistance by RTK inhibition with crizotinib *in vivo*

**Figure 7:**
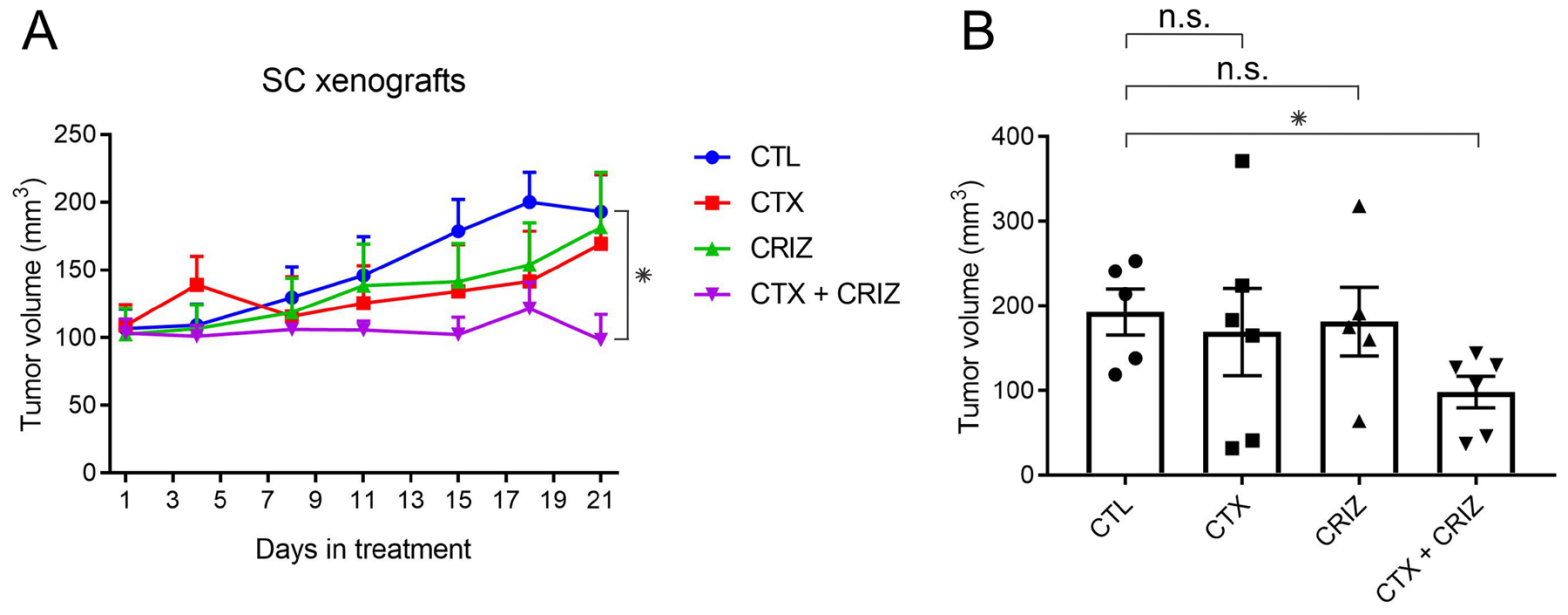
Overcoming cetuximab resistance by RTK inhibition with crizotinib *in vivo* (**A**) Five million SC cells were injected subcutaneously in female athymic nude mice. After the tumors reached an average size of 100 mm^3^, they were separated into four groups and treated with cetuximab (CTX, 0.5 mg/kg) and/or crizotinib (CRIZ, 5 mg/kg) as indicated for 21 days (*n* = 5 for CTL, *n* = 6 for CTX, CRIZ, and CTX/CRIZ). All tumors were measured twice a week, and average volumes for each group are plotted with SEM as corresponding error bars. ^*^indicates statistically significant differences (*p* < 0.05). (**B**) Graphical representation of the end point (day 21) volume measurements for each group as mean ± SEM. ^*^indicate statistically significant differences (*p* < 0.05). n.s. indicates no statistically significant difference between compared conditions (*p* ≥ 0.05).

We next tested if addition of crizotinib overcomes cetuximab resistance *in vivo*. For this experiment, we established SC subcutaneous xenografts in nude mice and treated them with cetuximab and crizotinib combinations for three weeks, monitoring subcutaneous tumor growth during this interval. We found that the cetuximab/crizotinib combination could inhibit SC subcutaneous xenograft growth, which neither drug was able to do individually (Figure 7A, 7B). These data indicate that the cetuximab/crizotinib combination provides a significant inhibitory effect *in vivo* in CRC xenografts.

## DISCUSSION

Advanced CRC management is particularly challenging where five-year survival rates fall below 15%. Cetuximab is approved by the U.S. FDA for advanced CRC, however, cetuximab is effective in less than 10% of selected cases, and resistance frequently arises [5–8, 18]. To better understand CRC and study the effect of targeted therapies, we employed our *in vitro* 3D CRC culture system that mimics *in vivo* behavior and can be manipulated in a fast and tractable fashion. As shown in Figure 1A, we established three lines from HCA-7 cells: CC (cetuximab sensitive), SC (*de novo* cetuximab resistant), and CC-derived CC-CR (acquired cetuximab resistant) [20, 21].

We previously showed that *de novo* cetuximab resistance in SC cells was associated with increased MET/RON phosphorylation and that MET/RON inhibition with crizotinib drastically reduced SC 3D colony growth in the presence of cetuximab [21]. For CC-CR cells, we previously showed that the mechanism of acquired resistance was due to upregulation of the long non-coding RNA MIR100HG and two embedded microRNAs, miR-100 and miR-125b, which upregulated the Wnt/β-catenin (WNT) signaling [20]. In this report, however, we show CC-CR cells additionally exhibit increased MET/RON phosphorylation compared to CC cells (Figure 1C and 1E). Thus, two cetuximab resistance mechanisms, increased WNT and RTK signaling, may operate in CC-CR. Consistent with this notion, cetuximab/crizotinib combination was less effective in reducing colony numbers in CC-CR compared to SC; ∼87% vs 98% reduction in CC-CR and SC colony numbers compared to their respective controls was observed (Figure 1B) [21]. CC-CR were similarly more resistant to panitumumab/crizotinib (Figure 2A, 2B) and MM-151/crizotinib (Figure 3A, 3B) combinations than SC. Whether the RTK- and WNT-dependent resistance mechanisms act independently or coordinately merits further investigation.

In this report, we show that multiple receptor tyrosine kinases are upregulated in SC and CC-CR cells, namely MET, RON, ERBB3, RET, and INSR (Figure 1D, 1E). These RTKs are implicated in CRC [16, 28–31]. Two or more of these RTKs may act in concert to achieve cetuximab resistance; they may introduce signaling redundancy and/or signaling diversity to achieve network robustness [32, 33]. Alternatively, RTK activity landscape may change as a function of tumor evolution. Consistent with the second possibility, we show that ERK1/2 signaling can be blocked by cetuximab in CC cells, while in cetuximab-resistant SC and CC-CR cells, ERK1/2 signaling can be blocked by crizotinib (Figure 1C). These results suggest that, upon acquiring cetuximab resistance, the downstream ERK1/2 signaling in SC and CC-CR cells is uncoupled from upstream EGFR activity and is instead governed by other RTKs, MET and RON.

We next tested the broader applicability of parallel inhibition strategy of inhibiting multiple RTKs simultaneously in CRC. We showed that multiple EGFR-directed therapies (cetuximab, panitumumab, and MM-151) and several multi-RTK inhibitors (crizotinib, cabozantinib, and BMS-777607) can act cooperatively to overcome *de novo* and acquired resistance in CRC (Figures 2, 3, 4). Panitumumab is a viable alternate to cetuximab, which in the Southeastern United States is associated with higher risk of allergic reactions [34]. MM-151 is a cocktail of three antibodies targeting distinct epitopes in EGFR extracellular domain and has been shown to be effective against EGFR extracellular domain mutations [26]. On the multi-RTK inhibitor side, BMS-777607 is a more selective MET family inhibitor, while bemcentinib selectively targets AXL, which might partially explain the cooperativity of the former with EGFR-directed therapies (Figure 4) [27, 35]. Although MET amplification, detected in peripheral blood, has been reported as a mechanism of resistance to EGFR inhibition, our work now shows increased MET activity, independent of its amplification, in both *de novo* and acquired modes of cetuximab resistance in CRC [16, 36, 37]. Genetic manipulation of MET/RON levels by shRNA or CRISPR-based methodologies is needed to confirm the role of individual RTKs.

One major strength of our 3D culture system is availability of distinct morphological and drug resistance phenotypes and their interconvertibility. We previously showed that spiky, filled lumen morphology of SC cultures can be converted to CC-like morphology, a uniform epithelial layer surrounding a hollow central lumen, by incubation with integrin beta 1 activating antibody P4G11 [38]. In this study, we showed that cetuximab-sensitive CC cells could be converted to cetuximab-resistance phenotype by addition of MET or ERBB3 ligands, HGF or NRG1, respectively (Figure 5A, 5B). While EGFR inhibition rescue by HGF addition in CRC has been shown before, a similar role for NRG1 addition has not been described [16, 39]. We additionally showed that HGF-induced cetuximab resistance could be overcome by crizotinib in CC 3D cultures (Figure 5C). Thus, availability of RTK ligands, which act in autocrine and paracrine manners, may influence cancer progression. Consistent with this notion, HGF, which is present in high levels in the preferred CRC metastatic site, liver, might act as a niche factor for CRC spread or resistance [40]. Similarly, NRG1 is also reported to express in liver [41]. Thus, RTK amplification and mutation do not comprise the whole cancer spectrum, instead recording RTK activity (by western blot analysis or RTK array) may integrate additional inputs emanating from ligand availability.

We showed that cetuximab and crizotinib cooperate to induce cell cycle arrest in G0/G1 phase in both SC and CC-CR cells (Figure 6). Future studies are needed to determine if the cell cycle block observed, upon longer incubation, leads to apoptosis induction; this may be measured by Annexin V-based FACS quantification, sub-G0 population, or PARP cleavage by western blot analysis.

All our studies in this report were performed in 3D, however, only a few cell lines were used; additional experiments need to be performed with more CRC cell lines in 3D and/or in patient-derived CRC xenografts and organoids. These efforts may further include other cancers that exhibit resistance to EGFR-directed therapies, like head and neck squamous cell carcinoma (HNSCC) [20]. Another limitation of the current study is the bulk analysis of the samples. Analysis at the single-cell level by new techniques such as multiplex immunofluorescence (MxIF) may inform tumor heterogeneity and help predict resistance emergence.

We finally tested the cetuximab/crizotinib combination *in vivo* in SC nude mice xenografts. In Figure 7A, we showed reduction in tumor growth in the combination treatment, whereas individual treatments were not significantly different than the untreated SC xenografts. Complimentary to previous reports of targeting MET amplification in cetuximab resistance, our work shows that MET activity is upregulated in *de novo* and acquired forms of cetuximab resistance and can be effectively targeted with multi-RTK inhibitors both *in vitro* and *in vivo* [16, 21]. In a previous attempt of overcoming cetuximab resistance in CRC with small molecule inhibitors, the MEK inhibitor pimasertib has been shown to delay onset of cetuximab resistance [42]. Since MEK is downstream of EGFR, this EGFR/MEK combined inhibition was termed vertical suppression. In another example, EGFR targeting is combined with downstream PI3K/AKT signaling in CRC [43]. By that analogy, our strategy of inhibiting multiple RTKs may be considered a parallel inhibition. Future studies may compare the relative effectiveness of these strategies and/or identify key regulatory determinants where one inhibition strategy is preferable over the other. In summary, we conclude that parallel inhibition strategy of multiple RTKs overcomes resistance to EGFR-directed therapeutics in CRC *in vitro* and *in vivo*.

## MATERIALS AND METHODS

### Cell culture

SC and CC cells were maintained in DMEM supplemented with 10% bovine growth serum, nonessential amino acids, L-glutamine, and penicillin/streptomycin. CC-CR were maintained in the above medium in the presence of cetuximab (3 µg/ml), which was removed during experiments in 3D.

### 3D type I collagen culture

Collagen was used at 2 mg/mL diluted in DMEM containing 10% fetal bovine serum. Typically, in each well of a 12-well dish, three layers of type I collagen, 400 ul each, were layered on top of another, with middle layers containing cells as a single-cell suspension. For typical colony counting assays, cells were seeded at a concentration of 5,000 cells/ml (2,000 cells/well). After setting of the three layers, 400 ul of medium was added to each well and was changed every two to three days. CC and SC colonies were typically counted at ∼14 days, while CC-CR colonies were counted at ∼20 days. Colonies were counted using GelCount (Oxford Optronix) with identical acquisition and analysis settings and represented as mean (*n* = 3) ± s.e.m. For western blotting experiments, 250,000 cells/ml (100,000 cells/well) were seeded in the middle layer. The cells were then stimulated a week later for indicated periods of time and lysed, processed, and subjected to immunoblotting.

### Reagents

PureCol bovine type I collagen was purchased from Advanced Biomatrix, Inc. (San Diego, CA, USA, #5005-100 ml). Unless specified otherwise, all cell culture components were purchased from Hyclone Laboratories, Inc. (Omaha, NE, USA). DMEM was purchased from Corning Mediatech (Manassas, VA, USA, #10-017-CV). Crizotinib was purchased from MilliporeSigma (Temecula, CA, USA, #PZ0191). Cabozantinib (#S1119), BMS-777607 (#S1561), and R428 (BGB324, #S2841) were purchased from Selleck Chemicals, Houston, TX, USA. Collagenase, type I was purchased from MilliporeSigma (Temecula, CA, USA, #234153). Recombinant human HGF was purchased from R&D Systems (Minneapolis, MN, USA, #294-HG/CF). Propidium iodide was purchased from Invitrogen (Carlsbad, CA, USA, #P3566).

### Antibodies

Primary antibodies used for western blot analysis were the following: anti-pERK1/2 (Cell Signaling #9101 rabbit antibody, 1:1,000); anti-ERK1/2 (Cell Signaling #9102 rabbit antibody, 1:1,000); anti-pAKT-serine473 (Cell Signaling #9271 rabbit antibody, 1:1,000); anti-AKT (Cell Signaling #9272 rabbit antibody, 1:1,000); anti-pMET-tyrosine1234/1235 (Cell Signaling #3077 rabbit antibody, 1:1,000); anti-MET (R&D #AF276 goat antibody, 1:2,000); anti-pRON-tyrosine1238/1239 (R&D #AF1947 rabbit antibody, 1 µg/ml); anti-RON (β C-20) (Santa Cruz #SC-322 rabbit antibody, 0.5 µg/ml); anti-tubulin (Calbiochem #CP06 mouse antibody, 1:5000). Secondary antibodies used were the following: goat anti-mouse (IRDye IgG H+L P/N 926-68020, LI-COR, 1:15,000); donkey anti-goat (IRDye IgG H+L P/N 926-68024, LI-COR, 1:15,000); donkey anti-rabbit (IRDye IgG H+L P/N 926-32213, LI-COR, 1:15,000); mouse TrueBlot ULTRA (anti-mouse IgG HRP, #18-8817-33, Rockland Inc., 1:2,000); rabbit TrueBlot (anti-rabbit IgG HRP, #18-8816-33, Rockland Inc., 1:2,000); goat TrueBlot (anti-goat IgG HRP, #18-8814-33, Rockland Inc., 1:2,000).

### Cell lysis, immunoprecipitation, and immunoblotting

The cell-containing middle collagen layer was removed and placed into 600 μL lysis buffer and incubated for 2 hours at 4°C while rotating. Lysis buffer composition: 50 mM HEPES (pH 7.5), 150 mM NaCl, 1% Triton X-100, 1 mM EDTA, 10% glycerol, and 10 mM sodium pyrophosphate. 2 mM sodium orthovanadate, 10 mM sodium fluoride, 1 mM PMSF, 5 μg/ml leupeptin, 5 μg/ml pepstatin and 5 μg/ml aprotinin were added fresh just before lysis. Lysates were precleared by centrifugation at 14,000 rpm for 15 min. Supernatants were diluted with 2x Laemmli sample buffer containing 5% β-mercaptoethanol, boiled for 5 min, resolved on SDS/PAGE, and electro-blotted onto nitrocellulose membranes. All blots were developed using chemiluminescence or LI-COR Odyssey.

### Phospho-RTK array

Cells were seeded in 3D as described above, lysed, and protein concentration in each sample was measured by the Micro BCA protein assay kit (Thermo Scientific #23235). Three hundred milligrams of protein were analyzed using the Human Phospho-Receptor Tyrosine Kinase Array Kit (R&D Systems, ARY001B) according to the manufacturer’s protocol. Array membranes were incubated with chemiluminescence detection solution, exposed to HyBlot CL autoradiography film (Thomas Scientific #E3012), and developed using a Kodak X-Omat processor (Kodak).

### FACS analysis

100,000 cells were seeded in 3D type I collagen cultures. Cells were allowed to grow for 1 week and incubated with indicated drugs for 20 hours. Middle layers containing cells were then removed and incubated with 1 ml of collagenase (3 mg/ml in DMEM) for 30 min at 37°C with gentle rocking. After this incubation, 300 μl of 10X trypsin and 15 μl of 0.5 mM EDTA were added and samples incubated under similar conditions for another 30 min. Cells were harvested by spinning at 200 *g* for 10 min at 4°C; they were then resuspended in 400 μl PBS by passing through a 25 gauge needle 5–7 times. Cells were fixed by adding 5 ml of 70% ethanol (–20°C) dropwise while vortexing and stored overnight at 4°C. Fixed cells were spun down at 1000 *g* for 10 min at 4°C, re-suspended in 500 μl PBS (containing 50 μl of 1 mg/ml propidium iodide and 10 μl of 10 mg/ml RNase A), and incubated in Eppendorf tubes rocking at 37°C for 1 hr. Samples were stored overnight at 4°C and then subjected to FACS analysis using BD™ LSRII (BD Biosciences, San Jose, CA) and analyzed with BD FACSDiva software (ver. 8.0.1).

### Statistics

Each experiment was performed at least three times in triplicates for each condition unless indicated otherwise. Statistical analyses were performed using GraphPad Prism software (version 7.02; GraphPad Software, Inc.). Significance determination was performed by ordinary one-way ANOVA followed by Tukey’s multiple comparisons test, with single pooled variance; *p* < 0.05 was considered statistically significant between the pairs tested.

### Nude mice injections and treatments

All procedures complied with the Guide for the Care and Use of Laboratory Animal Resources (1996), National Research Council, and were approved by the Vanderbilt University Institutional Animal Care and Use Committee. Five to six week old athymic nude mice were purchased from Charles Rivers Laboratories (Wilmington, MA). After one week of acclimatization, mice were subcutaneously injected with five million SC cells resuspended in 100 μl of sterile PBS. For approximately five weeks, SC xenografts were allowed to grow to an average volume of 100 mm^3^, at which stage the mice were randomly assigned to four groups with comparable average tumor burden. Cetuximab was given at a dose of 0.5 mg/kg (in PBS) via intraperitoneal injections every third day. Crizotinib was administered at a dose of 5 mg/kg (in water) by oral gavage every day. Tumor volume, animal weight, and health were recorded twice a week. The treatment lasted for three weeks. Tumor dimensions were measured by calipers, and volume was calculated using the formula volume = width × length × (height/2).

## SUPPLEMENTARY MATERIALS FIGURES AND TABLE


