# Supplementary material for: Broad-spectrum receptor tyrosine kinase inhibitors overcome de novo and acquired modes of resistance to EGFR-targeted therapies in colorectal cancer
Source: Oncotarget. 2019 Feb 12;10(13):1320–33. doi: 10.18632/oncotarget.26663 (PMC6407678; doi:10.18632/oncotarget.26663)
Supplement: Supplementary file 1 [file oncotarget-10-1320-s001.pdf]

Broad-spectrum receptor tyrosine kinase inhibitors overcome *de novo* and acquired modes of resistance to EGFR-targeted therapies in colorectal cancer

SUPPLEMENTARY MATERIALS

REF											REF
ERBB1	ERBB2	ERBB3	ERBB4	FGFR1	FGRR2A	FGFR3	FGFR4	INSR	IGF-1R	AXL	DTK
MER	MET	RON	PDGFRA	PDGFRB	SCFR	FLT-3	M-CSFR	RET	ROR1	ROR2	TIE1
TIE-2	TRKA	TRKB	TRKC	VEGFR1	VEGFR2	VEGFR3	MUSK	EPHA1	EPHA2	EPHA3	EPHA4
EPHA6	EPHA7	EPHB1	EPBH2	EPBH4	EPHB6	ALK	DDR1	DDR2	EPHA5	EPHA10	
REF		EPHB3	RYK								PBS

Supplementary Figure 1: Schematic of phospho-RTK array indicating location of the RTKs on the membrane. REF = reference spots (positive control), PBS = phosphate-buffered saline (negative control).

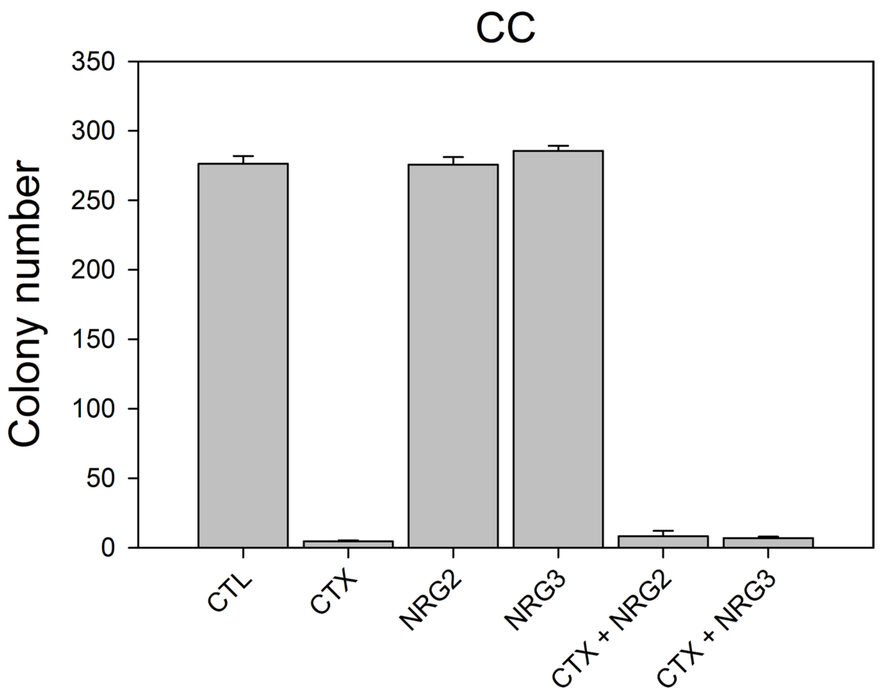

Supplementary Figure 2: Effect of NRG2, NRG3 on imparting cetuximab resistance to CC cells. Two thousand CC cells were cultured in type I collagen for two weeks. Fresh medium was added with cetuximab (CTX, 3 µg/ml), NRG2 (50 ng/ml), or NRG3 (50 ng/ml) every two to three days as indicated. Colony counts are plotted as mean ± SEM.

Supplementary Table 1: Therapeutic antibodies approved for the treatment of colorectal cancer

Antibody	Trade name	Company	Target	Type	FDA approval
Cetuximab	Erbitux	Eli Lilly, Merck, BMS	EGFR	Chimeric IgG1	2004
Bevacizumab	Avastin	Genentech (Roche)	VEGF	Humanized IgG1	2004
Panitumumab	Vectibix	Amgen	EGFR	Human IgG2	2006
Ramucirumab	Cryamza	Eli Lilly	VEGF	Human IgG1	2015
